# Enhanced activations in syntax-related regions for multilinguals while acquiring a new language

**DOI:** 10.1038/s41598-021-86710-4

**Published:** 2021-03-31

**Authors:** Keita Umejima, Suzanne Flynn, Kuniyoshi L. Sakai

**Affiliations:** 1 Department of Basic Science, Graduate School of Arts and Sciences, The University of Tokyo, 3-8-1 Komaba, Meguro-ku, Tokyo, 153-8902 Japan; 2 Department of Linguistics and Philosophy, Massachusetts Institute of Technology, 77 Massachusetts Avenue, Cambridge, MA 32-D80802139 USA

**Keywords:** Language, Cognitive neuroscience, Learning and memory

## Abstract

The neuroscientific foundation of multilingualism, a unique cognitive capacity, necessitates further elucidation. We conducted an fMRI experiment to evaluate the acquisition of syntactic features in a new language (Kazakh) for multilinguals and bilinguals. Results showed that the multilinguals who were more proficient in their second/third languages needed fewer task trials to acquire Kazakh phonology. Regarding group differences, the reduction in response times during the initial exposure to Kazakh were significantly larger for the multilinguals than the bilinguals. For the multilinguals, activations in the bilateral frontal/temporal regions were maintained at a higher level than the initial level during subsequent new grammar conditions. For the bilinguals, activations in the basal ganglia/thalamus and cerebellum decreased to the initial level each time. Direct group comparisons showed significantly enhanced activations for the multilinguals in the left ventral inferior frontal gyrus. These results indicate that both syntax-related and domain-general brain networks were more enhanced for the multilinguals. We also unexpectedly observed significant activations in the visual areas for the multilinguals, implying the use of visual representation even when listening to speech sounds alone. Because the multilinguals were able to successfully utilize acquired knowledge in an accumulated manner, the results support the cumulative-enhancement model of language acquisition.

## Introduction

Bilingualism—more generally, multilingualism—is a unique and commonplace achievement for most of the world’s population^1^, but how we acquire multiple languages is still not well understood, and the neuroscientific foundation of this process needs to be further elucidated. In this paper we argue that learning new languages is a cumulative process, and evidence for the *cumulative-enhancement model* proposed in this paper derives from fourth language acquisition of Kazakh in a syntactic task performed by Japanese first language (L1) learners, who have learned English as a second language (L2) and then learned a third language (L3), typically Spanish.

Decades of behavioral research have raised profound issues concerning the acquisition of multiple languages. The majority of these studies have focused on the differences and similarities between L1 and L2 acquisition. Namely, is it possible that the biologically determined language faculty, which has been argued to characterize and determine L1 acquisition, also significantly influences L2 acquisition as well? Traditional accounts, such as contrastive analysis^2,3^, the fundamental difference hypothesis^4–6^, connectionist proposals^7^, description of the dynamic systems theory^8^, and emergentist accounts^9^ have argued that this is not the case. However, there is an equally large, if not larger, body of evidence, that argues that L1 and L2 acquisition are fundamentally the same^10–18^. This body of research argues that the same principles that constrain the L1 acquisition process also constrain the L2 bilingual acquisition process.

More recently, empirical studies have focused on the degree to which L3 acquisition follows from the same principles that characterize both L1 and L2 acquisition. That is, to what degree can a theory of a domain-specific genetic endowment for language, which has been argued to characterize L1 and L2 acquisition, also characterize L3 acquisition? Do arguments, for example, about “the poverty of stimulus” still hold for multilingualism? Results indicate that L3, multilingualism, is constrained as in L1 acquisition and L2 bilingualism. However, the L3 learners have knowledge available to them about other languages (i.e., L1 and L2) that can be drawn upon in some way in the construction of a new language (i.e., L3) specific grammar. In fact, results indicate that more linguistic knowledge may in fact enhance the L3 acquisition process, and that therein lies a marked difference between L2 and L3 acquisition. It is revealed that L3 acquisition is constrained in the same way as L1 and L2, but there are more options available to the L3 learner. Such is the case as reported in a wide range of studies in adult L3 acquisition^19–21^. These behavioral results for L3 acquisition have led researchers to hypothesize that the more languages a learner knows, the easier it gets^22,23^.

Neuroscientifically, however, we are less informed concerning the neural substrates that might lead to differences in acquisition between L2 and L3 acquisition. There have been fewer neuroscience studies with respect to bilingualism and multilingualism. Moreover, previous studies have tended to be heavily defined by language pathologies, speech motor realizations of language, and cortical representations and organizations^24,25^. To our knowledge, no studies exist that evaluate the online, in vivo processing and acquisition of two or more languages. Based on the cumulative-enhancement model, we hypothesize that multilinguals [those who had acquired more than two languages (L1, L2, L3, …)] would exhibit more enhanced activations in the language-related regions than bilinguals [those who had acquired only two languages (L1, L2)], especially when a new target language is introduced.

We have previously shown that the most critical regions in syntactic processes are the left opercular/triangular parts of the inferior frontal gyrus (L. IFG) and left lateral premotor cortex (L. LPMC), which are involved in both L1 and L2 acquisition^26–28^. A possible *universal* function of the L. IFG could be a syntactic process called Merge, i.e., a simple and primitive combinatory operation to create a phrase or clause^29,30^. Activations in this region actually predicted “the Degree of Merger,” i.e., the maximum depth of merged subtrees (called Mergers), in a sentence; the more binary syntactic nodes (or branches) the sentence had, the more active the region became^31,32^. It has been reported that there were differential activations when participants were exposed to *natural* versus artificial rules of sentences^33,34^. We showed localized activation in the left language areas including the L. IFG under the Natural condition, while under the Artificial condition, activation was observed in the bilateral LPMC, together with wide-spread regions^34^. In the present study of natural languages, we examined how the deep properties of sentence constructions (e.g., coordination and subordination) were generalized for the bilinguals and multilinguals. More specifically, they may utilize acquired knowledge in an accumulated manner, as expected to be reflected by neuronal signatures of these critical regions. We also predict increased activations in the bilateral auditory areas that are critical for listening to new sounds, as well as subcortical regions and the cerebellum required by adaptive controls for changes in sounds or linguistic rules. The left caudate activation has been reported when proficient bilinguals switched languages during semantic judgements with words^35^. Activations in these regions would be maintained throughout exposures to a new language for multilinguals, who were capable of manipulating multiple languages in a rapid and precise manner. In addition, the cerebellum has been proposed to control not only motor but mental activities by encoding internal models^36^. We expect associated changes in activations in these regions along with the acquisition processes of a new language, where multilinguals would exhibit more enhanced activations than bilinguals.

In the present study with functional magnetic resonance imaging (fMRI), we recruited participants, who were all native speakers of Japanese and had acquired English as their L2. We divided the participants into two groups according to their listening scores on the language proficiency tests (the Avant tests graded by scores 1–9; see Supplementary Methods). One group was a *bilingual* (Bi) group (N = 21) consisting of participants who had scores of 2 or higher in their L2 (English) while scores in L3 were 1 if any. The other group was a *multilingual* (Multi) group (N = 28) consisting of participants who had Avant scores of 2 or higher in both their L2 (English) and L3 (predominately Spanish) (see Tables S1 and S2). We used Kazakh as a new target language to acquire. As a member of the Kipchak branch within Turkic, Kazakh is a native language in Kazakhstan and the neighboring countries. Kazakh is a head-final and agglutinative language with a modifier-head word order, and with an SOV word order for declarative sentences^37^. These syntactic parameters are shared with Japanese, but the Japanese language happens to lack the agreement of grammatical features, including a parameter of subject-verb agreement^38^. Because subject-verb agreement is present in Kazakh, just as in English and Spanish, we tested how this “hidden” parameter, as well as associated syntactic processes, was acquired by Japanese participants who had linguistic knowledge of English and/or Spanish.

**Figure 1:**
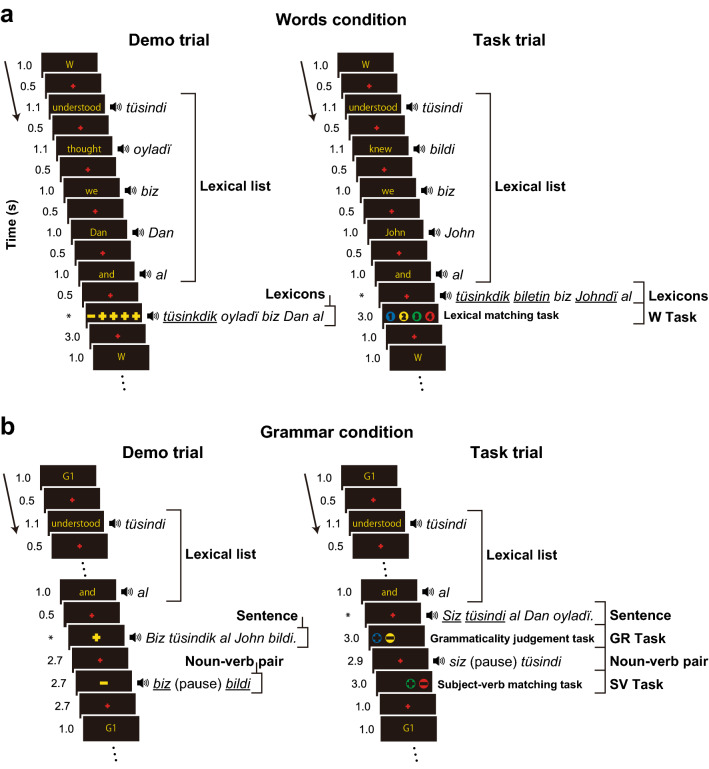
Words and Grammar conditions in Kazakh. (**a**) A trial under the Words (W) condition. A set of five Kazakh words (Lexicons) followed the Lexical list, replacing some words in the Lexical list with ones having an inflectional change, but in the same order (underlined words denote mismatch). In the demo trials, the +/− signs indicated correctness of simultaneously presented stimuli, while in the task trials, the participants chose a number (or a sign under the Grammar conditions) for the stimuli presented immediately before. Asterisks denote the variable duration of an auditory presentation. (**b**) A trial under the Grammar condition of G1. A Kazakh sentence (Sentence; underlined words denote ungrammaticality) followed the Lexical list, after which a noun–verb pair was presented (underlined words denote mismatch). The example sentence in Kazakh shown in the demo trial means “*We understood and John knew*,” while that in the task trial is ungrammatical. For the task trials, there was a silent pause of 0.5 s between the Lexical list and Sentence/Lexicons. In activation analyses, we focused on the temporal events of the Sentence, Lexicons, and Lexical list in the task trials. These images were created using Adobe Illustrator (ver. 16.0.3, https://www.adobe.com/products/illustrator.html).

All the participants were acquiring Kazakh for the first time, and we used basic Kazakh words (see Table S3). We made the acquisition of syntactic knowledge to be a progressive stepwise process under three Grammar conditions, in which more complex syntactic structures were gradually introduced: G1, G2, and G3 (see Table S4). We developed a grammaticality judgement task (GR Task) and a subject-verb matching task (SV Task) for each of the Grammar conditions, as well as using a lexical matching task for control (W Task) under the Words condition (Fig. 1). In each trial, five Kazakh words ("Lexical list") were auditorily presented, each with a translated word in English on the display. We alternated *demo* with *task* trials under each of the Words and Grammar conditions. Under the Grammar conditions, the first sign (+ or −) indicated correctness (i.e., grammaticality) of the sentence, while the second sign showed correctness of the noun–verb pairing in the sentence in a *demo* trial. In a *task* trial without such clues, the participants were asked to judge those and to press a colored button (+ or −) after each presentation in Kazakh. Using this novel experimental design, critical differences between the multilinguals and bilinguals could be properly elucidated.

## Results

### Relationships among multiple language abilities for the Multi group

**Figure 2:**
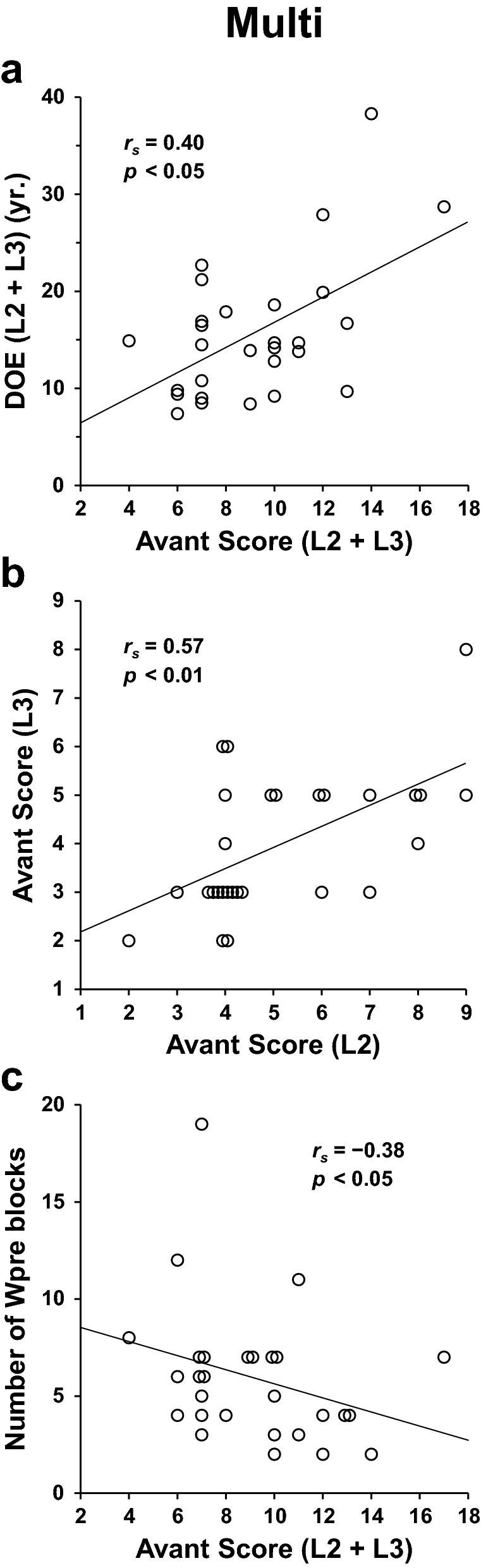
Consistency among multiple language abilities for the multilingual (Multi) group. (**a**) A significant correlation between the Avant scores (a standard listening proficiency test) and duration of exposure (DOE), where the scores were combined for second and third languages (L2 and L3). (**b**) Correlation between the Avant scores in L2 and L3. (**c**) The negative correlation between the combined Avant scores and number of blocks (eight trials per block) to reach the criterion for Wpre (“preparatory” task trials under the Words condition). These results indicate consistent listening abilities among multiple languages of L2, L3, and Kazakh (initial exposure). The horizontally spreading dots were expanded manually to show multiple discrete points. These images were created using Microsoft Excel 2013 (ver. 15.0.5319.1000, https://www.microsoft.com) and Adobe Illustrator (as above).

For the Multi group, who showed large individual differences in experience and proficiency in both their L2 and L3, we assessed their duration of exposure (DOE; experience) and Avant scores (proficiency levels) in detail. The DOE was significantly longer in their L2 than in their L3 (paired *t*-test, *t*(27) = 13 , *p* < 0.0001), and the Avant scores were significantly higher in their L2 than in their L3 (*t*(27) = 3.9, *p* = 0.0005) (Table S1). Moreover, as shown in Fig. 2a, the combined Avant scores in their L2 and L3 correlated with the combined DOE in their L2 and L3 (Spearman’s rank correlation test, *r*_*s*_ = 0.40, *p* = 0.04).

As indicated in Fig. 2b, we also found a positive correlation between the Avant score in their L2 and that in their L3 (*r*_*s*_ = 0.57, *p* = 0.002), even when the differences between their L2 and L3 in DOE, and those in ages of acquisition (*t*(27) = 13, *p* < 0.0001), were significant. The combined Avant scores in their L2/L3 could then be correlated with task performances in Kazakh as well. Indeed, we observed a negative correlation (*r*_*s*_ =  −0.38, *p* = 0.04) between the combined Avant scores and the number of blocks under the Words condition (Wpre), which were *preparatory* to the Grammar conditions (Fig. 2c; see Supplementary Methods). This significant correlation was replicated for the L2 scores alone (*r*_*s*_ =  −0.38, *p* = 0.05), but not for the L3 scores alone (*r*_*s*_ =  −0.26, *p* = 0.2). In summary, the participants with higher listening proficiencies in their L2/L3 adapted to the inflectional changes in Kazakh faster.

### Group differences in the progressive acquisition of Kazakh grammars

**Figure 3:**
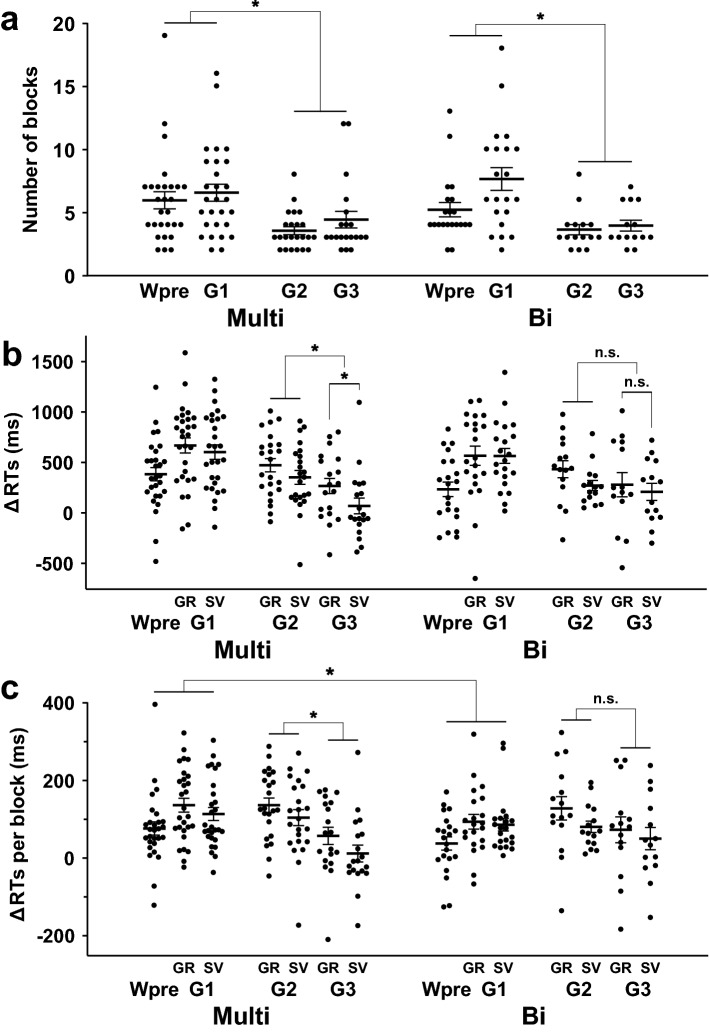
Group differences in the progressive acquisition of Kazakh grammars. (**a**) The mean numbers of blocks needed for the participants to reach criterion under each condition, together with individual data points overlapped. The progressive acquisition of Kazakh grammars was observed from Wpre/G1 to G2/G3 for both Multi and bilingual (Bi) groups. (**b**) The reduction in RTs (∆RTs) from the first to last blocks. Significant differences were observed between G2 and G3, as well as between GR and SV Tasks under G3, only for the Multi group. (**c**) ∆RTs per block, showing a significant group difference under the Wpre/G1 conditions. Error bars indicate standard errors of the mean (SEM). *p < 0.05; n.s., not significant (*p* > 0.1). These images were created using GraphPad Prism (ver. 9.0.1, https://www.graphpad.com) and Adobe Illustrator (as above).

The criterion for mastering each task was to perform correctly in at least six out of the eight task trials in each of two blocks. Under each of the G1-G3 conditions, this passing criterion must have been met for both GR and SV Tasks. We examined the number of blocks required to reach the criterion (Fig. 3a), and this number was comparable between the Multi and Bi groups under each condition (*t*-test, *p* > 0.3). Therefore, individual variances in the number of runs cannot explain group differences in behavioral results or brain activations. Nonetheless, there was a significant decrease in the number of blocks from Wpre/G1 to G2/G3 (Multi, *t*(49) = –3.5, *p* = 0.001; Bi, *t*(34) = –3.4, *p* = 0.002), importantly indicating the progressive acquisition of Kazakh grammars for both groups.

In addition, response times (RTs) were also comparable between the groups for the first and last blocks under all conditions (Wpre/G1/G2/G3, *p* > 0.05). We next evaluated the *reduction* in RTs from the first to last blocks (i.e., ∆RTs) for each task (Fig. 3b). We observed significant decreases in ∆RTs from G2 to G3 only for the Multi group (*t*(40) = –3.0, *p* = 0.005), as well as significant differences between GR and SV Tasks under G3, i.e., between G3-GR and G3-SV (paired *t*-test, *t*(18) = 2.2, *p* = 0.04). These results indicate that acquired knowledge for G2 was successfully transferred to G3, where the SV Task became less demanding than the GR.

To reveal any dynamic changes in RTs during the progressive acquisition, we examined ∆RTs divided by the number of blocks (i.e., ∆RTs per block). Consistent with the results of ∆RTs, ∆RTs per block showed a significant decrease from G2 to G3 (*t*(40) = –3.6, *p* = 0.001), only for the Multi group (Fig. 3c). The Bi group showed a similar tendency, but individual differences became larger. Moreover, a repeated-measures ANOVA (rANOVA) on ∆RTs per block with factors of group [Multi, Bi] and task [Wpre, G1-GR, G1-SV] showed both main effects of group (*F*(1, 47) = 4.3, *p* = 0.04) and task (*F*(2, 94) = 7.6, *p* = 0.0009), without an interaction (*F*(2, 94) = 0.1, *p* = 0.9). Because ∆RTs per block was larger for the Multi group than the Bi group, the Multi group showed a larger improvement during the initial exposure to Kazakh (Wpre/G1). Individual rANOVAs on later G2-GR/G2-SV or G3-GR/G3-SV showed neither main effects (*p* > 0.05) nor any interactions (*p* > 0.5).

### Activation in the lateral regions for the Multi and Bi groups

We analyzed the six critical blocks (i.e., MR scanning runs under the Grammar conditions), namely the first run (an *initial* state) and last run (a *final* state) under each of the G1–G3 conditions: G1 first, G1 last, G2 first, G2 last, G3 first, and G3 last. To examine whether there were any activation patterns unique to each Grammar condition, we combined the first and last runs under each of G1–G3 (e.g., G1 first and G1 last).

**Figure 4:**
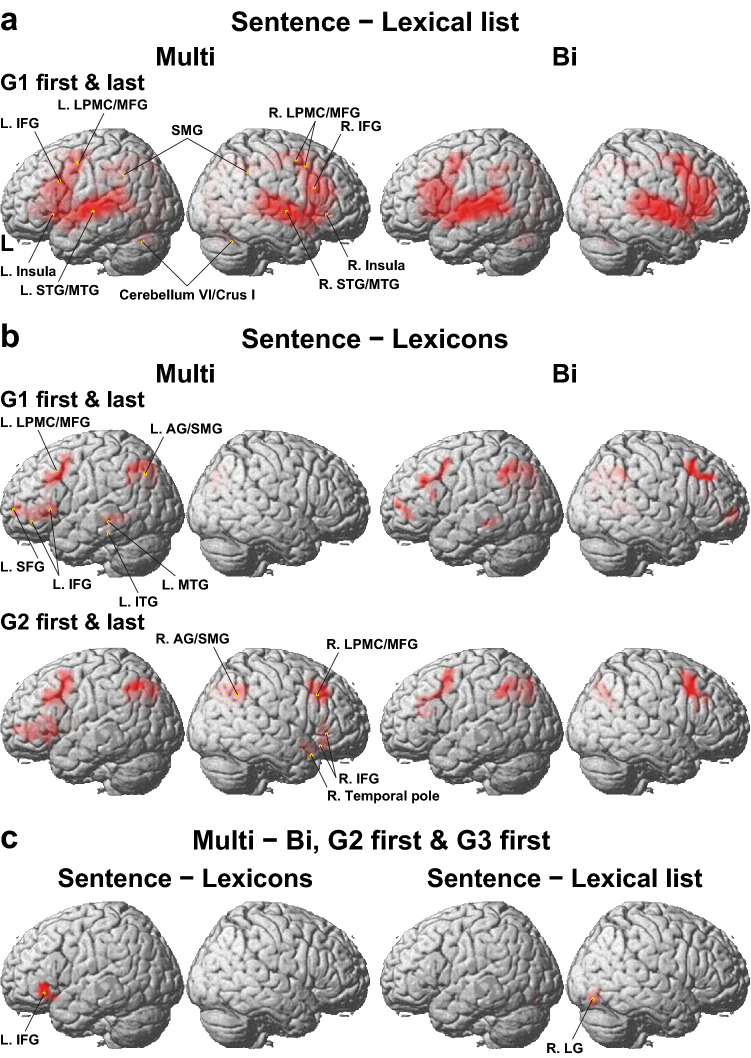
Activation in the lateral regions for the Multi and Bi groups. (**a**) Results of the Sentence–Lexical list contrast in task trials, where "Sentence" and "Lexical list" refer to the the particular events of those presented in Fig. 1. This contrast is shown for the first and last runs under G1 averaged (i.e., *G1 first* and *G1 last*, respectively). Both groups showed significant bilateral activations in the frontal and temporal regions (FWE corrected *p* < 0.05 for the cluster level). (**b**) Results of the more stringent Sentence–Lexicons contrast, where "Sentence" and "Lexicons" refer to the the particular events of those presented in Fig. 1. This contrast is shown for G1 (first and last) and G2 (first and last). For each condition, an exclusive mask of negative activation for the Lexicons were applied (one-sample *t*-test, uncorrected *p* < 0.005). (**c**) A direct comparison between the two groups (i.e., Multi–Bi), averaged for the G2 first and G3 first runs. Activations were shown for the Sentence–Lexicons contrast (left panel), and for the Sentence–Lexical list contrast (right panel). Each yellow dot indicates the local maximum of activated regions on the lateral surface (see Table S5). These images were created using SPM12 software (Wellcome Trust Center for Neuroimaging, http://www.fil.ion.ucl.ac.uk/spm) and Adobe Illustrator (as above).

We first tested the “Sentence–Lexical list” contrast, i.e., a comparison between two types of stimuli: sentences and the list of words in the same task trials under the Grammar conditions ("Sentence" and "Lexical list" refer to the particular events of those presented in Fig. 1). The Lexical list controlled common basic auditory and encoding processes of words against the Sentence. Activation patterns in the lateral views were all similar under each Grammar condition, such that the most prominent activations were observed bilaterally in the LPMC/ middle frontal gyrus (LPMC/MFG) and IFG, as well as the superior/middle temporal gyri (STG/MTG) (Fig. 4a; see Table S5 for the list of activated regions for the Multi group). These activation patterns were also similar between the two groups.

Next, we examined the more stringent “Sentence–Lexicons” contrast, i.e., a comparison between two types of stimuli: sentences under the Grammar conditions, and the list of words (always with inflectional changes) under the Words condition ("Sentence" and "Lexicons" refer to the the particular events of those presented in Fig. 1). The Lexicons strictly controlled auditory and memory retrieval processes against the Sentence. During G1 first and G1 last, the Multi group showed left-lateralized activation in the LPMC/MFG, ventral IFG, superior frontal gyrus (SFG), MTG, inferior temporal gyrus (ITG), and angular/supramarginal gyri (AG/SMG) (Fig. 4b, Table S5). In contrast, the Bi group showed bilateral activations in the LPMC and AG/SMG, as well as in a bilateral region more anterior to the inferior frontal sulcus (BA 10/46). During G2 first and G2 last, the Multi group showed activations in the following right regions in addition to the above frontal and parietal regions: the right LPMC/MFG, ventral IFG, temporal pole, and AG/SMG. In contrast, in the Bi group, the activation patterns did not change significantly between the G1 and G2 runs (see Fig. 4b). Note that significant activations were absent in the ventral frontal regions for the Bi group, which was an additional indication of group differences.

Because there was no significant difference in activation between the groups during G1, G2, or G3, we examined additional combinations of two runs (e.g., G1 first and G2 first). In the Sentence–Lexicons contrast, we found significant activations in the “Multi–Bi” contrast only when the G2 first and G3 first runs were combined (Fig. 4c, Table S5). Significant activation was localized in the left ventral IFG alone, confirming the results of Fig. 4b. Moreover, in the Sentence–Lexical list contrast with the same combination of runs, significant activation was observed in the right lingual gyrus (R. LG). These results demonstrate activation increases selective to the Multi group. The explanation of why such increases were observed for G2 first and G3 first will be clarified by subsequent analyses of these individual runs.

### Differential subcortical activation between the Multi and Bi groups

**Figure 5:**
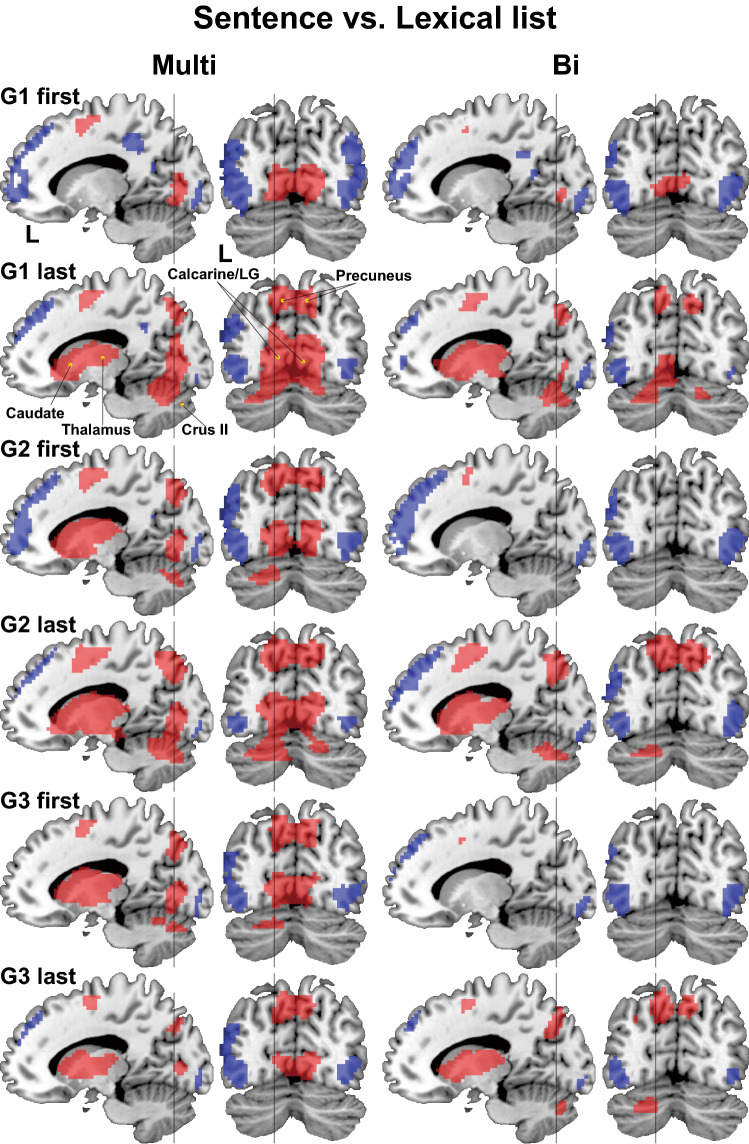
Differential activation in the other cortical and subcortical regions between the Multi and Bi groups. Activated regions for the Sentence–Lexical list contrast are shown in red, while those for Lexical list–Sentence are shown in blue (FWE corrected *p* < 0.05 for the cluster level). Parasagittal sections at *x* = −15 in MNI coordinates showed subcortical activations (red), whereas coronal sections at *y* = −72 showed occipital activations (red). The vertical lines indicate locations of those sections. Each yellow dot indicates the local maximum of activated regions on the medial structures (see Table S5). The Multi group showed significant activations in the basal ganglia/thalamus for G1 last, which maintained throughout the subsequent runs. Moreover, localized activations were observed in the visual cortex for the Multi group consistently during all of the runs. These images were created using SPM12 software (as above), MRIcron software (ver. 6.6.2013, https://www.nitrc.org/projects/mricron), and Adobe Illustrator (as above).

Contrary to the activation patterns in the lateral views in the Sentence–Lexical list contrast (Fig. 4a), the *subcortical* regions revealed marked group differences (Fig. 5, parasagittal sections, shown in red). The basal ganglia/thalamus showed significant activation for the Multi group, which was persistent for G1 last and subsequent runs. Note that activation patterns between G1 last and G2 first, as well as those between G2 last and G3 first, were almost identical. In contrast, for the Bi group, the basal ganglia/thalamus activations alternated between first and last runs under the G1–G3 conditions, which can be characterized as “renewing cycles” adapting to the new grammatical conditions. In other words, activations observed for G1 last and G2 last disappeared almost completely for G2 first and G3 first, respectively. The left cerebellum also replicated the same alternating pattern of activation (Fig. 5, *coronal* sections, shown in red). These differential patterns of activations cannot be explained by the experimental procedures, because both groups received an equivalent number of blocks under all conditions (Fig. 3a), and because inter-session intervals for the transition from G1 to G2, and that from G2 to G3, were mostly within the same day or the day after (median: 1 day) without significant differences (Wilcoxon rank sum test, W = 169, *p* = 0.9).

**Figure 6:**
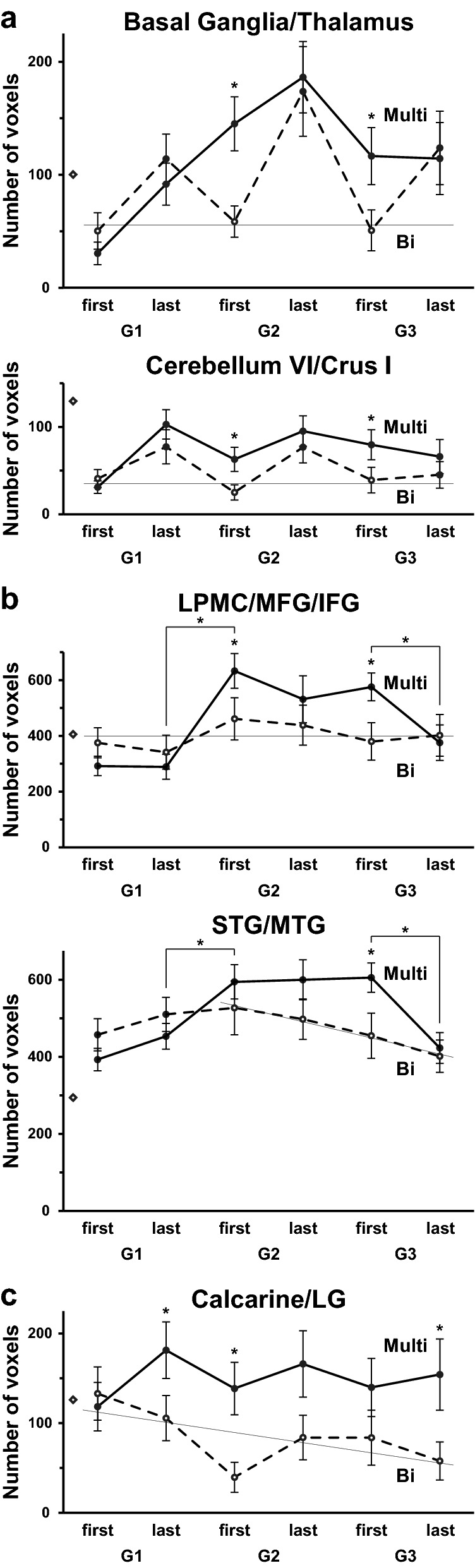
Temporal patterns of activations for the Multi group distinct from those for the Bi group. Within each ROI, a number of activated voxels (estimated by the contrast of Sentence–Lexical list, uncorrected *p* < 0.001 for each participant) was counted and shown for each run. The number of voxels for a ROI in each hemisphere was treated as independent samples. For each panel, a thin fitting line for selected open points of the Bi group is shown, in accordance with individual temporal patterns of activations. The rhombus symbols on the ordinate denote 10% voxels within each ROI, where the scale of each graph was normalized with this fixed ratio. (**a**) The number of voxels in the basal ganglia/thalamus and in the cerebellum. (**b**) The number of voxels in the lateral premotor cortex/middle/inferior frontal gyrus (LPMC/MFG/IFG) and in the superior/middle temporal gyri (STG/MTG), i.e., the language-related regions and right homologs. (**c**) The number of voxels in the calcarine/lingual gyrus (calcarine/LG). Error bars indicate SEM. *p < 0.05. These images were created using Microsoft Excel 2013 and Adobe Illustrator (as above).

To show statistical differences in activations between the Multi and Bi groups, we quantified regional activations by counting the number of activated voxels (above the threshold of uncorrected *p* < 0.001) within a region of interest (ROI) for each participant. In the basal ganglia/thalamus (Fig. 6a), the numbers of voxels for the Bi group increased during each of the G1–G3 conditions and returned to the ground level (G1 first) between the conditions, but for the Multi group, the numbers persisted above the ground level from G1 last until G3 last. The numbers for the Multi group were significantly higher for G2 first and G3 first compared to those for the Bi group (G2 first: one-tailed *t*-test, *t*(74) = 2.7, *p* = 0.004; G3 first: *t*(64) = 2.0, *p* = 0.03). In the cerebellum (VI/Crus I), the temporal patterns of activations were basically replicated, and the same group differences were observed (G2 first: *t*(74) = 2.0, *p* = 0.02; G3 first: *t*(64) = 1.7, *p* = 0.05). These results indicate that the modulatory systems such as the subcortical regions and cerebellum adapted to sentences of a new language, such that those sentences were processed de novo for G2 first and G3 first for the Bi group, while these renewing cycles became less obvious for the Multi group.

### Enhanced activations in the frontal and temporal regions for the Multi group

As regards the LPMC/MFG/IFG activation (Fig. 6b), the numbers of voxels for the Bi group stayed at initial level (G1 first) for all runs. However, for the Multi group, the numbers became higher than the initial level from G2 first until G3 first, showing significantly higher numbers for G2 first and G3 first compared to the Bi group (G2 first: *t*(74) = 1.7, *p* = 0.04; G3 first: *t*(64) = 2.4, *p* = 0.01). In the STG/MTG, the numbers for the Bi group gradually declined from G2 first to G3 last, possibly due to habituation with the auditory stimuli. In contrast, the numbers remained high from G2 first until G3 first for the Multi group, showing significantly higher numbers for G3 first than those for the Bi group (*t*(64) = 2.2, *p* = 0.01). Notably, these results indicate more enhanced syntactic or auditory processes for the Multi group.

Regarding the temporal patterns of activations for the Multi group, we found a significant *increase* in the number of voxels between G1 last and G2 first in both of the frontal and temporal regions (LPMC/MFG/IFG: *t*(100) = 4.5, *p* < 0.0001; STG/MTG: *t*(100) = 2.6, *p* = 0.006). Because some participants did not meet passing criteria for G2 or G3, we performed unpaired *t*-tests here. Moreover, we observed a significant *fall* in activations during G3 first and G3 last in these regions (LPMC/MFG/IFG: *t*(74) = –2.5, *p* = 0.008; STG/MTG: *t*(74) = –3.3, *p* = 0.0007). These results indicate that the temporal patterns of activations in the language-related regions and right homologs for the Multi group were completely different from those in the basal ganglia/thalamus and cerebellum for the Bi group (Fig. 6a,b).

**Figure 7:**
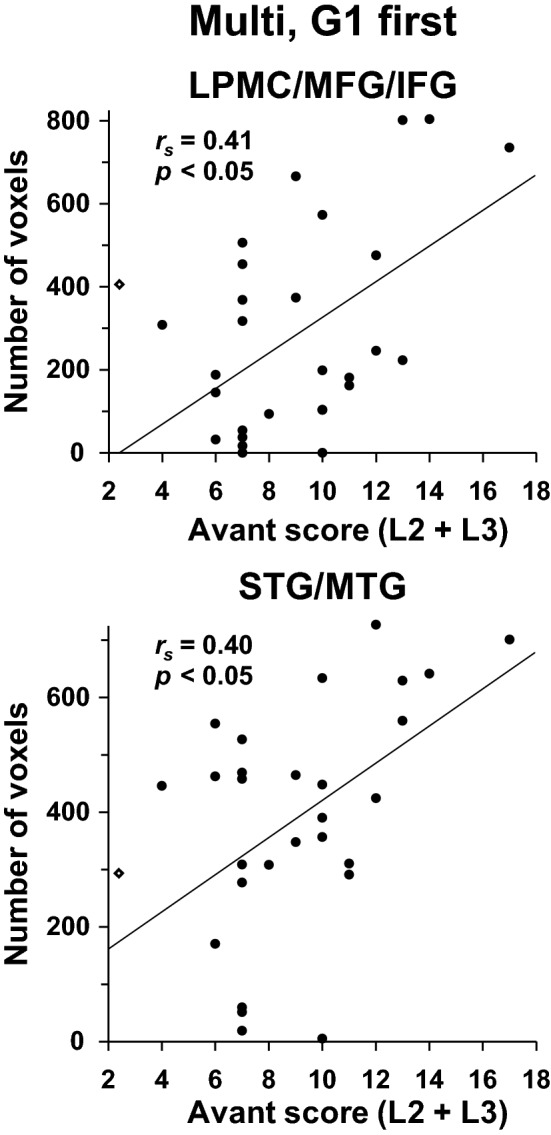
Activations for G1 first predicted by language proficiency of the Multi group. The numbers of voxels in the LPMC/MFG/IFG (**a**) and STG/MTG (**b**), averaged for the ROI in both hemispheres of each participant of the Multi group showed a significant correlation with the combined Avant scores. The rhombus symbols on the ordinate denote 10% voxels within each ROI, as shown in Fig. 6. These images were created using Microsoft Excel 2013 and Adobe Illustrator (as above).

We examined whether activations in the language-related regions and right homologs reflect individual differences in language proficiency. We focused on the initial exposure to Kazakh sentences, i.e., for G1 first. We observed significant correlations between the combined Avant scores and the numbers of voxels for the Multi group (Fig. 7; LPMC/MFG/IFG: *r*_*s*_ = 0.41, *p* = 0.03; STG/MTG: *r*_*s*_ = 0.40, *p* = 0.04). Such correlations were not significant for the other runs (|*r*_*s*_|< 0.3, *p* > 0.3). These results indicate that the more proficient in multiple languages the participants were, the more the syntactic and auditory processes were enhanced, especially during the initial exposure to sentences.

### Stimuli-independent and persistent activation in visual areas for the Multi group

In the Sentence–Lexical list contrast for the Multi group (Fig. 5), we unexpectedly observed activation in the visual areas throughout all runs (coronal sections, shown in red). This activation remained, even if we subtracted out the events of the Lexical list when visual stimuli were also presented, and in spite of the lack of visual stimuli during the event of the Sentence (see Fig. 1b). More specifically, the anterior (i.e., *rostral*) part of the calcarine/LG, corresponding to more peripheral regions (i.e., from perifovea to macula) of the visual field, showed significant activation (see the parasagittal sections shown in red). As regards the Bi group, in contrast, the activation in the rostral calcarine/LG was significant during G1 first and G1 last alone, and was much weaker than the Multi group. To identify activation due to *real* visual stimuli, we then examined the *reversed* contrast of Lexical list–Sentence (Fig. 5, shown in blue), where the Lexical list involved the visual presentation of translated words in English (see Fig. 1b). Visual areas were clearly shown with this contrast, including the posterior (i.e., *caudal*) part of the calcarine/LG, corresponding to more central regions (i.e., from fovea to parafovea) of the visual field (see the parasagittal sections, shown in blue). These results indicate the involvement of visual areas even when listening to speech sounds alone.

In the calcarine/LG (Fig. 6c), the numbers of voxels for the Bi group declined throughout the runs. In contrast, for the Multi group, the numbers preserved the initial high level until the last run of G3 last. The numbers for the Multi group were significantly higher for G1 last, G2 first, and G3 last, compared to those for the Bi group (G1 last: *t*(96) = 1.8, *p* = 0.04; G2 first: *t*(74) = 2.5, *p* = 0.006; G3 last: *t*(64) = 1.9, *p* = 0.03). These results statistically confirmed more enhanced and maintained activations for the Multi group in the visual areas.

## Discussion

All languages acquired can potentially influence the development of subsequent language learning in a positive manner. This claim contrasts sharply with deficit models of language learning, in which negative transfer/interference or the role of non-linguistic/cognitive factors are the hallmarks of the development of multilingualism. We hypothesized that the deep properties of sentence constructions, such as coordination and subordination, would be internalized and thus become a fundamental basis of language when multiple languages have been acquired in a natural manner.

Selective activations in the bilateral LPMC/MFG/IFG and STG/MTG were observed in the present study (Fig. 4a), as well as those in the left ventral IFG for the Multi group (Fig. 4c), suggesting more enhanced syntactic processes^32^ and/or engagement of syntactic knowledge. These syntactic roles in the left frontal regions were supported by the right frontal regions among the three syntax-related networks^39^, whereas bilateral temporal activations may reflect enhanced attention to sound patterns^40^. Moreover, during the initial phase of L2 acquisition, activations in the left LPMC/MFG/IFG indeed increased when language proficiency became higher^28,41^. In the present study, the right frontal regions were recruited by the multilinguals when more complex syntactic structures were required under G2, whereas the bilinguals showed right frontal activations under both G1 and G2 (Fig. 4b). Even though we used a limited set of words and tested syntactic features like subject-verb agreement, the number of their combinations became infinite, thus requiring the acquisition of syntactic knowledge as opposed to explicit rule and pattern learning in general. This syntactic knowledge is crucially underlain by Merge-generability^34^, the key concept of human language, which is in marked contrast with artificial rules in general.

We have previously indicated that “cortical activations increase initially at the onset of acquisition, followed by the maintenance of the activations and then a fall in activations during consolidation of linguistic competence^26^.” It is striking to note that the multilinguals, but not the bilinguals, showed the same pattern of activations during the short period of this experiment (Fig. 6b). This pattern also explains the group difference in the left ventral IFG (Fig. 4c), because the G2 first and G3 first were the end points of the maintenance phase. Moreover, at the initial phase of G1 first, the numbers of voxels in the language-related regions and right homologs positively correlated with proficiency in L2/L3 for the Multi group (Fig. 7). It is thus likely that the processes of acquiring linguistic/syntactic rules and new sound patterns were initially facilitated and later reduced for the multilinguals.

An fMRI study with a path-finding task reported activations in the bilateral striatum and cerebellum under the exploratory conditions with unknown rules, as well as under the model-based conditions with pre-learned rules^42^. Similar to such exploratory and model-based conditions, the consecutive G1–G3 conditions also involved exploration of unknown rules in a new language. We observed selective activations in the basal ganglia/thalamus and cerebellum in the contrast of Sentence–Lexical list (Figs. 5, 6a), further indicating that the multilinguals better coordinated the language-related regions and domain-general adaptive control systems than the bilinguals.

Regarding the calcarine/LG activation (Figs. 4c, 5, 6c), the LG is a part of the network processing syntax and input/output interface together with the left LPMC, left angular gyrus, and cerebellar nuclei^39^, in which activations were replicated in the present study (Fig. 4b). The recruitment of the right LG, as well as the left ventral IFG, the main syntax-related region, in the multilinguals may indicate deeper processing of syntactic structures. By using the double contrasts of “Sentence–Lexical list” and “Lexical list–Sentence,” we demonstrated that the rostral and caudal parts of the calcarine/LG were separately activated, suggesting the use of peripheral vision even during the auditory presentation of a sentence, especially for the multilinguals. A possible non-linguistic account for calcarine/LG activations is the use of visual imagery (e.g., an array of words or a scene for the sentence)^43^, in spite of strict sensory and attentional controls between the Sentence and Lexical list. Our results further suggest that the multilinguals are able to utilize multimodal (auditory-visual here) information even during language use.

## Materials and methods

For more details, see the Supplementary Methods.

### Participants

Volunteers (62 native Japanese speakers) were openly recruited from multiple sources, including the LEX Institute (Hippo Family Club), the University of Tokyo, and Sophia University, as well as personal invitation from the participants. All the participants were acquiring Kazakh for the first time in the experiment. We did not exclude any participant unless there was a definite, compelling reason. One participant had both Japanese and Turkish as her L1; that participant was included because the effect of dual L1 was unknown and she was not exceptionally faster in meeting criteria for Wpre/G1/G2 compared with other multilinguals. Right-handedness was estimated as a laterality quotient (LQ) according to the Edinburgh inventory^44^, and two participants were eliminated from the analyses because of their left-handedness (i.e., negative LQ), and one because of such likelihood (LQ: 6.7). Another participant was eliminated whose Avant score in English was 1. Nine participants were eliminated because they did not reach the criterion even for G1.

The resultant 49 participants were all right-handed (LQ: 47–100); none of them had psychiatric disorders. As regards the Multi group, 28, 23, and 20 participants passed G1, G2, and G3, respectively; one participant was excluded from analyses under G3 due to head movement in the scanner. Regarding the Bi group, 21, 15, and 14 participants passed G1, G2, and G3, respectively. Once the participants acquired the simplest structure under G1 (i.e., coordination of two simple sentences), it would generally have become easier for them to understand more complex syntactic structures under G2/G3.

Between the Bi and Multi groups, there was no significant difference in age (*t*-test, *t*(47) = 1.6, *p* = 0.1) or laterality quotient (LQ) (*t*(47) = 1.7, *p* = 0.09). Whereas the DOE to the L2 significantly differed between the groups (*t*(47) = 2.2, *p* = 0.03, longer for the Multi), Avant scores in their L2 were comparable (*t*(47) = 1.4, *p* = 0.2). Although the Bi group had experienced their L3 primarily at universities, their listening abilities in L3 were minimal (see Table S2). In contrast, most members of the Multi group achieved 3 or higher for the Avant scores in their L3. Therefore, the major difference between the groups was whether or not they had acquired an L3.

Prior to their participation in the study, the nature and possible consequences of the study were explained to each participant and written informed consent was obtained afterwards. Approval for the experiments was obtained from the institutional review board from the University of Tokyo, Komaba (No. 464). All research was performed in accordance with the Declaration of Helsinki, Singapore Statement on Research Integrity, and relevant guidelines/regulations in Japan (Science Council of Japan, and Japan Society for the Promotion of Science). This clinical trial has been registered in a publically accessible primary register at Japan Registry of Clinical Trials (jRCT) on 25/12/2020 (No. jRCT1030200294).

### Stimuli

Auditory stimuli in Kazakh consisted of 146 sentences (80 grammatical and 66 ungrammatical) constructed with the words shown in Table S3, which were recorded by a male native speaker of Kazakh. Both grammatical and ungrammatical sentences were articulated at a somewhat slower pace, and individual words were also separately recorded. By using the Wavelab 8 software (Steinberg Media Technologies GmbH, Hamburg, Germany), we digitized the stimuli (16 bit, 44.1 kHz, stereo), where the maximum volume of each stimulus was equally set to −1 dBFS. We tried to arrange the sentence stimuli to a fixed length as much as possible, maintaining their original pitches; their duration was adjusted to 4.3 s, 4.2 s, and 4.65 s under the G1, G2, and G3 conditions with different function words, respectively. During the scans, the participants wore an MRI-compatible headphone (Resonance Technology Inc., Northridge, CA), a pair of earmuffs (3 M Peltor, St. Paul, MN), and a pair of earplugs (Earasers, Persona Medical, Casselberry, FL) to reduce the high-frequency noises (> 1 kHz) of the scanner.

### Tasks

Under the Words condition (see Fig. 1), a row of five signs (+ or −), in situ from left to right, indicated correctness of matching between the Lexical list and a set of five Kazakh words in a *demo* trial. In a *task* trial without such clues, the participants were asked to count the number of mismatched words, and to press a colored button for that number. Regarding the Grammar conditions, see main text (the Introduction).

Under both the Words and Grammar conditions, a block of four *demo* trials and a block of eight *task* trials were alternately administered. Depending on how quickly they completed the tasks, participants received 30–104 blocks (220–704 trials) combining all Words and G1–G3 conditions (e.g., a G1 run contained a block of eight G1 trials, as well as five Words trials). In the demo trials, Kazakh words were presented to the participants under the Words condition in order to acquire knowledge of the sound patterns, whereas Kazakh sentences were presented under the Grammar conditions in order to acquire knowledge of the syntactic structures. Throughout the experiments, the participants never received explicit explanations about the syntactic rules or about sentence structures in Kazakh. After each block of task trials, the participants always received feedback on their number of correct responses (e.g., 6 out of 8), separately for GR and SV Tasks under each Grammar condition. Different sets of stimuli were used for the demo and task trials to avoid judgements that had been simply memorized by the participants.

### MRI data acquisition

The MRI scans were conducted in a 3.0 T scanner (Signa HDxt; GE Healthcare, Milwaukee, WI) with a bird-cage head coil. Each participant was in a supine position, and his or her head was immobilized inside the coil. As regards the structural images, high-resolution T1-weighted images of the whole brain [136 axial slices, 1 × 1 × 1 mm^3^] were acquired with a three-dimensional fast spoiled gradient-echo (3D FSPGR) acquisition [repetition time (TR) = 8.6 ms, echo time (TE) = 2.6 ms, flip angle (FA) = 25°, field of view (FOV) = 256 × 256 mm^2^]. With respect to the time-series data of fMRI, we used a gradient-echo echo-planar imaging (EPI) sequence [TR = 2 s, TE = 30 ms, FA = 78°, FOV = 192 × 192 mm^2^, resolution = 3 × 3 mm^2^]. We scanned a set of 30 axial slices that were 3-mm thick with a 0.5-mm gap, covering the range of −38.5 to 66 mm from the line of the anterior commissure to posterior commissure (AC-PC). In a single scanning session, we obtained 145 volumes, and dropped the initial four volumes from analyses due to MR signal increases.

The fMRI data were analyzed in a standard manner using SPM12 statistical parametric mapping software (Wellcome Trust Center for Neuroimaging, http://www.fil.ion.ucl.ac.uk/spm)^45^ implemented on MATLAB (Math Works, Natick, MA). A random-effects analysis for a group was performed with statistical thresholds of uncorrected *p* < 0.001 for the voxel level, and family-wise error (FWE) corrected *p* < 0.05 for the cluster level.

## Supplementary Information

Supplementary Information.

## Data Availability

The datasets generated during and/or analyzed during the current study are available from the corresponding author on reasonable request.
