# Supplementary material for: Enhanced activations in syntax-related regions for multilinguals while acquiring a new language
Source: Sci Rep. 2021 Mar 31;11:7296. doi: 10.1038/s41598-021-86710-4 (PMC8012711; doi:10.1038/s41598-021-86710-4)
Supplement: Supplementary file 1 — Supplementary Information. [file 41598_2021_86710_MOESM1_ESM.docx]

**Enhanced Activations in Syntax-Related Regions for Multilinguals While Acquiring a New Language**

**Keita Umejima^1^, Suzanne Flynn^2^, and Kuniyoshi L. Sakai^1,*^**

^1^ Department of Basic Science, Graduate School of Arts and Sciences, The University of Tokyo, 3-8-1 Komaba, Meguro-ku, Tokyo 153-8902, Japan

^2^ Department of Linguistics and Philosophy, Massachusetts Institute of Technology,

77 Massachusetts Avenue, 32-D808, Cambridge, MA 02139, USA

*Correspondence: Department of Basic Science, Graduate School of Arts and Sciences, The University of Tokyo, Komaba, 3-8-1 Komaba, Meguro-ku, Tokyo 153-8902, Japan. Fax: +81-3-5454-6261. *E-mail*: sakai@sakai-lab.jp (K.L. Sakai).

**Table S1. Basic data for participants, whose first language (L1) was Japanese.**

| Group | N | Age (yr.) | L2 | | |  | L3 | | | LQ |
| --- | --- | --- | --- | --- | --- | --- | --- | --- | --- | --- |
|  |  |  | AOA (yr.) | DOE (yr.) | Avant score |  | AOA (yr.) | DOE (yr.) | Avant score |  |
| Bilinguals  (Bi) | 21  (10 f.) | 20.0 ± 3.1  (14.9-26.3) | 11.1 ± 2.8  (2.5-14.1) | 8.9 ± 3.5  (3.2-16.0) | 4.5 ± 1.4  (2-9) |  | − | − | ≤ 1 | 86 ± 15  (47-100) |
| Multilinguals  (Multi) | 28  (18 f.) | 21.3 ± 2.5  (16.2-27.1) | 9.8 ± 3.0  (0.0-13.0) | 11.6 ± 4.6  (6.1-27.1) | 5.2 ± 1.9  (2-9) |  | 17.1 ± 1.7  (12.8-21.6) | 4.2 ± 2.9  (0.2-11.2) | 4.0 ± 1.4  (2-8) | 93 ± 13  (60-100) |

For each factor, averaged data (mean ± standard deviation) and their ranges (in parentheses) are shown. The Avant score shows language proficiency assessed by the listening test of the Avant STAMP 4S (scores 1-9). N: number of participants, L2: second language, L3: third language, AOA: age of acquisition, DOE: duration of exposure, LQ: laterality quotient.

**Table S2. Language backgrounds of the participants.**

| Bilinguals (Bi) | | | | | | | | | | | | | | | | | | |
| --- | --- | --- | --- | --- | --- | --- | --- | --- | --- | --- | --- | --- | --- | --- | --- | --- | --- | --- |
| No. | Age | L2: English | | |  | L3 | | | |  | L4 | | | |  | L5 | | |
|  |  | DOE | DOR | Avant |  | Lg. | DOE | DOR | Avant |  | Lg. | DOE | DOR | Avant |  | Lg. | DOE | DOR |
|  |  | (yr.) | (mo.) | score |  |  | (yr.) | (mo.) | score |  |  | (yr.) | (mo.) | score |  |  | (yr.) | (mo.) |
| 1 | 20.5 | 10.1 |  | 2 |  | ZH | 2.1 |  |  |  |  |  |  |  |  |  |  |  |
| 2 | 19.4 | 7.2 |  | 4 |  | DE | 0.1 |  |  |  |  |  |  |  |  |  |  |  |
| 3 | 18.9 | 7.1 |  | 4 |  |  |  |  |  |  |  |  |  |  |  |  |  |  |
| 4 | 18.6 | 6.2 |  | 4 |  |  |  |  |  |  |  |  |  |  |  |  |  |  |
| 5 | 16.5 | 5.1 |  | 4 |  | FR | 1.2 |  |  |  |  |  |  |  |  |  |  |  |
| 6 | 18.3 | 4.2 | 13 | 7 |  |  |  |  |  |  |  |  |  |  |  |  |  |  |
| 7 | 17.0 | 14.5 |  | 4 |  |  |  |  |  |  |  |  |  |  |  |  |  |  |
| 8 | 16.5 | 8.2 |  | 4 |  |  |  |  |  |  |  |  |  |  |  |  |  |  |
| 9 | 15.9 | 3.2 |  | 4 |  | ES | 0.2 |  | 1 |  |  |  |  |  |  |  |  |  |
| 10 | 19.2 | 8.3 |  | 4 |  | KO | 0.2 |  |  |  |  |  |  |  |  |  |  |  |
| 11 | 18.5 | 5.2 |  | 4 |  |  |  |  |  |  |  |  |  |  |  |  |  |  |
| 12 | 24.6 | 12.3 |  | 5 |  | DE | 6.3 |  |  |  |  |  |  |  |  |  |  |  |
| 13 | 14.9 | 4.3 |  | 4 |  |  |  |  |  |  |  |  |  |  |  |  |  |  |
| 14 | 26.3 | 13.8 |  | 5 |  | ZH | 7.9 |  |  |  |  |  |  |  |  |  |  |  |
| 15 | 23.6 | 11.0 |  | 4 |  | DE | 4.9 |  |  |  |  |  |  |  |  |  |  |  |
| 16 | 21.5 | 11.0 |  | 4 |  | DE | 1.9 |  |  |  | RU | 1.0 |  |  |  |  |  |  |
| 17 | 21.8 | 9.0 |  | 4 |  | IT | 3.0 |  |  |  |  |  |  |  |  |  |  |  |
| 18 | 22.5 | 9.9 |  | 9 |  | ES | 3.0 |  |  |  |  |  |  |  |  |  |  |  |
| 19 | 23.8 | 11.0 |  | 4 |  | ZH | 3.0 |  | 1 |  |  |  |  |  |  |  |  |  |
| 20 | 21.3 | 9.0 |  | 4 |  | ES | 3.0 |  |  |  |  |  |  |  |  |  |  |  |
| 21 | 20.7 | 16.0 |  | 6 |  | ZH | 2.0 |  |  |  |  |  |  |  |  |  |  |  |
| Multilinguals (Multi) | | | | | | | | | | | | | | | | | | |
| No. | Age | L2: English | | |  | L3 | | | |  | L4 | | | |  | L5 | | |
|  |  | DOE | DOR | Avant |  | Lg. | DOE | DOR | Avant |  | Lg. | DOE | DOR | Avant |  | Lg. | DOE | DOR |
|  |  | (yr.) | (mo.) | score |  |  | (yr.) | (mo.) | score |  |  | (yr.) | (mo.) | score |  |  | (yr.) | (mo.) |
| 1 | 18.3 | 7.6 |  | 3 |  | ES | 2.2 | 11 | 3 |  |  |  |  |  |  |  |  |  |
| 2 | 24.1 | 11.1 | 5 | 5 |  | PT | 5.7 | 10 | −* |  | ES | 3.1 | 9 | 5 |  |  |  |  |
| 3 | 25.3 | 19.4 | 4 yr. | 9 |  | ES | 9.3 | 11 | 8 |  |  |  |  |  |  |  |  |  |
| 4 | 18.5 | 12.5 |  | 5 |  | ES | 2.2 | 11 | 5 |  |  |  |  |  |  |  |  |  |
| 5 | 25.0 | 18.6 |  | 7 |  | ES | 9.3 | 10 | 5 |  | ZH | 5.6 | 4 | − |  |  |  |  |
| 6 | 20.2 | 11.7 |  | 4 |  | ES | 2.8 | 10 | 3 |  | KO | 0.7 |  | − |  |  |  |  |
| 7 | 22.4 | 11.6 | 10 | 4 |  | ES | 9.6 | 5 | 3 |  |  |  |  |  |  |  |  |  |
| 8 | 23.4 | 16.5 |  | 4 |  | ES | 6.2 | 11 | 3 |  |  |  |  |  |  |  |  |  |
| 9 | 22.0 | 13.6 |  | 4 |  | ES | 4.3 | 10 | 4 |  |  |  |  |  |  |  |  |  |
| 10 | 20.5 | 10.6 |  | 4 |  | ES | 3.3 | 11 | 5 |  | KO | 0.6 |  | − |  |  |  |  |
| 11 | 20.8 | 10.7 |  | 2 |  | ES | 4.2 | 9 | 2 |  |  |  |  |  |  |  |  |  |
| 12 | 21.4 | 8.6 |  | 6 |  | ES | 5.2 | 10 | 5 |  |  |  |  |  |  |  |  |  |
| 13 | 18.2 | 6.7 |  | 4 |  | ES | 2.3 | 10 | 3 |  |  |  |  |  |  |  |  |  |
| 14 | 23.3 | 10.6 |  | 4 |  | ES | 6.3 | 11 | 3 |  |  |  |  |  |  |  |  |  |
| 15 | 19.7 | 7.6 |  | 4 |  | ES | 3.2 | 10 | 3 |  |  |  |  |  |  |  |  |  |
| 16 | 23.2 | 12.6 | 2 | 8 |  | ES | 7.3 | 11 | 4 |  |  |  |  |  |  |  |  |  |
| 17 | 20.4 | 7.6 |  | 8 |  | ES | 2.1 | 9 | 5 |  |  |  |  |  |  |  |  |  |
| 18 | 22.4 | 13.9 | 11 | 6 |  | FR | 3.9 |  | − |  | ES | 0.8 | 9 | 5 |  |  |  |  |
| 19 | 19.5 | 7 |  | 4 |  | ES | 1.5 | 10 | 3 |  |  |  |  |  |  |  |  |  |
| 20 | 18.3 | 6.1 |  | 4 |  | ES | 1.3 | 10 | 2 |  |  |  |  |  |  |  |  |  |
| 21 | 27.1 | 27.1 | 4 yr. | 9 |  | ES | 11.2 |  | 5 |  | ZH | 7.6 |  | − |  |  |  |  |
| 22 | 22.5 | 14.9 |  | 4 |  | ES | 3.7 | 9 | 6 |  |  |  |  |  |  |  |  |  |
| 23 | 19.6 | 7.7 |  | 6 |  | ES | 0.7 |  | 3 |  |  |  |  |  |  |  |  |  |
| 24 | 20.0 | 12 | 10 | 8 |  | ES | 4.7 |  | 5 |  |  |  |  |  |  |  |  |  |
| 25 | 16.2 | 9.2 | 2 | 4 |  | ES | 0.2 |  | 2 |  |  |  |  |  |  |  |  |  |
| 26 | 19.8 | 7.1 |  | 4 |  | RU | 2.1 |  | 6 |  |  |  |  |  |  |  |  |  |
| 27 | 23.6 | 11.2 |  | 4 |  | DE | 5.3 |  | 3 |  | ES | 2.3 |  |  |  | ZH | 0.9 |  |
| 28 | 21.6 | 9.9 |  | 7 |  | DE | 2.9 |  | 3 |  | IT | 2.8 |  | − |  | ZH | 2.6 |  |

Second to fifth languages (L2-L5), if any, of each participant are listed separately for the Multi and Bi groups (see main text for definition). One participant in the Multi group (#21) had both Japanese and Turkish as L1. *Not tested in this study. DOR: duration of residence in countries where the language is spoken, DOE: duration of exposure, Lg.: language, DE: German, ES: Spanish, FI: Finnish, FR: French, IT: Italian, KO: Korean, PT: Portuguese, RU: Russian, ZH: Chinese.

**Table S3. Words in Kazakh used here.**

| Verbs | | | | Translation in English |
| --- | --- | --- | --- | --- |
| Past | Adjectival participle | | |  |
|  | Perfect | | Habitual past |  |
| *tüsindi* | *tüsingen* | | −* | *understood* |
| *oyladï* | *oylaɣan* | | − | *thought* |
| *tanïdï* | − | | *tanïytïn* | *recognized* |
| *bildi* | − | | *biletin* | *knew* |
| Nouns | | | |  |
| Nom. | | Acc. | |  |
| *biz* (first pl.) | | *bizdi* | | *we*/*us* |
| *siz* (second sg.) | | *sizdi* | | *you*/*you* |
| *ol* (third sg.) | | *onï* | | *he*/*him* |
| *John* | | *Johndï* | | *John*/*John* |
| *Dan* | | *Dandï* | | *Dan*/*Dan* |
| Function words | | | |  |
| *al* | | | | *and* |
| *dep* | | | | *that* |
| *kez* (nom.) | | *kezde* (loc.) | | *time*, *when* |
| *adam* (nom.) | | *adamdï* (acc.) | | *man*, *who* |

In our experiments, we used four verbs, five nouns (three pronouns and two proper nouns), and four function words in Kazakh. The verbs in past tense had suffixes in agreement with the person and number of the subject (subject-verb agreement). The polite form *siz* was used for a second singular person, and only the locative form *kezde* was used here. *Not used in this study. nom.: nominative, acc.: accusative, loc.: locative, sg.: singular, pl.: plural.

**Table S4. Sentence constructions under the Grammar conditions.**

| Grammar conditions | | Constructions and example sentences |
| --- | --- | --- |
| G1 |  | N_1_ V_1_ *al* N_2_ V_2_. |
| *al* condition | (1) | *Biz* *tüsindik al John oyladï*. “*We understood and John thought.*” |
|  | (2) | **Biz* *tüsindik al John oyladïŋïz*. |
| G2 |  | N_1_ N_2_ V_2_ *dep* V_1_. |
| *dep* condition | (3) | *Biz* *John oyladï dep tüsindik.* “*We understood that John thought.*” |
|  | (4) | **Biz* *John oyladï dep tüsindi.* |
| G3 |  | Type i: N_2_ V_2_ *kezde* N_1_ V_1_. |
| *kezde* condition | (5) | *John* *oylaɣan kezde biz tüsindik.* “*When John thought, we understood.*” |
|  | (6) | **John* *oyladï kezde biz tüsindik.* |
|  |  | Type ii: N_1_ V_1_, N_2_ V_2_ *kezde*. |
|  | (7) | *John oyladï,* *biz tüsingen kezde.* “*John thought when we understood.*” |
|  | (8) | **John oyladïq,* *biz tüsingen kezde.* |

The same index (1 or 2) denotes a noun (N) and a verb (V) in individual clauses, where the noun is a subject. A pair of grammatical and ungrammatical (*) sentences are shown for each Grammar condition (see Fig. 1). The verb inflection basically agrees with the subject’s person and number, except the verb just before *kezde*. Participants acquired such grammatical knowledge through demo trials without any explicit instruction.

**Table S5. Regions with activations selective to the sentence presentation.**

| Brain regions | BA | Side | *x* | *y* | *z* | *Z* | voxel |  | Brain regions | BA | Side | *x* | *y* | *z* | *Z* | voxel |
| --- | --- | --- | --- | --- | --- | --- | --- | --- | --- | --- | --- | --- | --- | --- | --- | --- |
| Multi, G1 first & last, Sentence – Lexical list | | | | |  |  |  |  | Multi, G2 first & last, Sentence – Lexicons | | | | |  |  |  |
| LPMC/MFG | 6/9 | L | –27 | –4 | 50 | Inf | 12459 |  | LPMC/MFG | 6/9 | L | –42 | 23 | 41 | 7.2 | 975 |
|  |  | R | 30 | –1 | 53 | 7.5 | * |  | IFG | 44/45 | L | –48 | 11 | 23 | 3.8 | * |
|  |  |  | 48 | 8 | 47 | 7.5 | * |  |  | 45 | L | –48 | 20 | 5 | 3.9 | * |
| IFG | 44/45 | L | –54 | 14 | 32 | Inf | * |  |  | 47 | L | –42 | 41 | –1 | 4.5 | * |
|  |  | R | 51 | 17 | 26 | Inf | * |  |  |  |  | –27 | 23 | –13 | 4.2 | * |
| Insula |  | L | –30 | 29 | –1 | Inf | * |  | SFG | 10/11 | L | –27 | 59 | 5 | 4.1 | * |
|  |  | R | 33 | 29 | –1 | Inf | * |  | ACC | 32 | L | –15 | 20 | 50 | 4.4 | * |
| SMA/ACC | 6/32 | M | 3 | 11 | 53 | Inf | * |  | LPMC/MFG | 6/9 | R | 36 | 20 | 38 | 5.1 | 359 |
| STG/MTG | 22/21 | L | –57 | –19 | 2 | Inf | * |  | ACC | 32 | R | 15 | 29 | 47 | 3.9 | * |
|  |  | R | 57 | –10 | 2 | Inf | * |  | IFG | 45 | R | 51 | 29 | –1 | 4.4 | 182 |
| SMG | 40 | L | –36 | –49 | 38 | 6.8 | * |  |  | 47 | R | 30 | 23 | –13 | 4.0 | * |
|  |  | R | 39 | –49 | 41 | 6.0 | * |  | Temporal pole | 38 | R | 54 | 14 | –22 | 4.6 | * |
| Caudate |  | M | –9 | 14 | 2 | 7.6 | * |  | AG/SMG | 39/40 | L | –45 | –61 | 41 | 7.2 | 1253 |
|  |  |  | 12 | 14 | 2 | Inf | * |  |  |  | R | 48 | –58 | 38 | 6.2 | * |
| Thalamus |  | M | 12 | –13 | 8 | 7.1 | * |  | Precuneus | 7 | M | –6 | –64 | 38 | 7.1 | * |
|  |  |  | –12 | –13 | 8 | 6.9 | * |  | Caudate |  | L | –15 | –1 | 20 | 3.7 | 1072 |
| Precuneus | 7 | M | –9 | –76 | 44 | 7.0 | 3540 |  |  |  | R | 15 | 11 | 23 | 3.6 | * |
|  |  |  | 9 | –73 | 44 | 6.7 | * |  |  |  | M | –9 | 20 | 2 | 5.2 | * |
| Calcarine/LG | 17/18/19 | M | –12 | –79 | 2 | Inf | * |  |  |  |  | 9 | 23 | 2 | 4.2 | * |
|  |  |  | 6 | –79 | –1 | Inf | * |  | Thalamus |  | M | 6 | –1 | –4 | 4.9 | * |
| Cerebellum VI/Crus I | | L | –27 | –67 | –28 | Inf | * |  | Vermis |  | M | 0 | –34 | –1 | 4.8 | * |
|  |  | R | 24 | –64 | –28 | 7.1 | * |  |  |  |  |  |  |  |  |  |
| Cerebellum Crus II | | M | –9 | –79 | –31 | 6.7 | * |  | Multi – Bi, G2 first & G3 first, Sentence – Lexicons | | | | | | | |
|  | |  |  |  |  |  |  |  | IFG | 47 | L | –45 | 29 | –7 | 4 | 104 |
| Multi, G1 first & last, Sentence – Lexicons | | | | | | | |  |  |  |  |  |  |  |  |  |
| LPMC/MFG | 6/9 | L | −48 | 17 | 44 | Inf | 584 |  | Multi – Bi, G2 first & G3 first, Sentence – Lexical list | | | | | | | |
| IFG | 45 | L | −51 | 23 | 5 | 5.1 | * |  | LG | 18 | R | 15 | –82 | –13 | 5.3 | 125 |
|  | 47 | L | −48 | 41 | −7 | 5.3 | * |  |  |  |  |  |  |  |  |  |
| SFG | 10/11 | L | −24 | 59 | 5 | 5.9 | * |  |  |  |  |  |  |  |  |  |
| MTG | 21 | L | −60 | −34 | −7 | 4.6 | 131 |  |  |  |  |  |  |  |  |  |
| ITG | 20 | L | −42 | −34 | −19 | 3.2 | * |  |  |  |  |  |  |  |  |  |
| AG/SMG | 39/40 | L | −39 | −70 | 41 | 7.6 | 239 |  |  |  |  |  |  |  |  |  |
| Precuneus | 7 | M | −6 | −64 | 38 | Inf | 555 |  |  |  |  |  |  |  |  |  |

Stereotactic coordinates (*x*, *y*, *z*) in the MNI space are shown for activation peaks of *Z* values, which were more than 16 mm apart in either direction of the *x*, *y*, or *z* axis (see Fig. 4, 5). The region with an asterisk is included within the same cluster shown in the nearest row above. The activations for the Bi group were generally subsets of those for Multi. Uncorrected *p* < 0.001 for the voxel level, family-wise error (FWE) corrected *p* < 0.05 for the cluster level. BA: Brodmann’s area; L: left, M: medial, R: right; ACC: anterior cingulate cortex, AG: angular gyrus, LG: lingual gyrus, LPMC: lateral premotor cortex, SFG/MFG/IFG: superior/middle/inferior frontal gyrus, SMA: supplementary motor area, SMG: supramarginal gyrus, STG/MTG/ITG: superior/middle/inferior temporal gyrus.

**Supplementary Methods**

***The Kazakh vocabulary used in this study***

Thirteen words (Table S3) were utilized, consisting of four verbs (*tüsindi* “*understood*,” *oyladï* “*thought*,” *tanïdï* “*recognized*,” and *bildi* “*knew*”), three pronouns (*biz* “*we*,” *siz* “*you*,” and *ol* “*he*”), two proper nouns (*John* and *Dan*), a conjunction (*al* “*and*”), a complementizer (*dep* “*that*”), and two common nouns (*kez* “*time*,” “*when*” and *adam* “*man*,” “*who*”). We translated *al* as “*and*,” instead of the actual meaning of “*meanwhile*” for the sake of simplicity. As regards *kez*, the locative form *kezde* was used. In Kazakh, the four words *al*, *dep*, *kezde*, and *adam* can be regarded as function words.

With respect to the verbs, we used the simple past tense form (“past” in short) like *tüsindi* and *tanïdï* (see Table S3, verbs), as well as an adjectival participle with inflectional changes: its perfect form *tüsingen*, and its habitual past form *tanïytïn*. As regards the nouns, their suffixes change according to their cases. An accusative form is made by adding the suffix allomorph -*di*/-*dï* to the nominative form (see Table S3, nouns), except for *ol* with the accusative form *onï*. With respect to the selection between -*di*/-*dï*, Kazakh suffix allomorphs are determined according to the rules of vowel harmony within a word.

In this experiment, we used the following syntactic subjects: the first person plural *biz*, second person singular (polite form) *siz*, and third person singular *ol*, *John*, and *Dan*. In addition, the verbs in the past tense take personal suffixes according to the person and number of the subject (i.e., subject-verb agreement). According to the rules of vowel harmony in Kazakh, the personal suffix of a verb becomes the allomorphs -*dik*/-*dïq* for the subject *biz* (first person plural), and -*diŋiz*/-*dïŋïz* for the subject *siz* (second person singular), while for the subject of a third person singular, the personal suffix of a verb becomes the allomorphs -*di*/-*dï*. As shown in Table S3, the adjectival participles always have a fixed suffix, irrespective of the person and number of a subject. All of these syntactic forms were checked and confirmed by native speakers of Kazakh.

***The Kazakh grammar used in this study***

Three Grammar conditions, namely G1, G2, and G3, were tested in consecutive order (Table S4). These conditions required identification of the syntactic structures of a sentence in Kazakh, involving all of the verbs, pronouns, and proper nouns listed above, while *al*, *dep*, and *kezde* were used only under the G1, G2, and G3 conditions, respectively. Under the G1 condition, two simple sentences with two nouns and two verbs (N_1_ V_1_, N_2_ V_2_) were coordinated with *al* (“*and*”) [example (1) in Table S4], and participants had to recognize the grammaticality of the subject-verb agreement first. Under the G2 condition, a simple sentence (N_2_ V_2_) was embedded in another one (N_1_ V_1_) [example (3) in Table S4], and participants recognized this structure based on the knowledge of the subject-verb agreement. Note that the complementizer *dep* (“*that*”) follows the embedded sentence (N_2_ V_2_), and that the verb (V_1_) of the main clause follows the embedded clause (N_2_ V_2_ *dep*) as an object clause (see Table S4, G2).

Under the G3 condition, the common noun *kezde* (“*when*”) is placed at the end of a subordinate (temporal) clause (N_2_ V_2_ *kezde*). We tested both sentence types of i) the subordinate clause followed by a main clause (N_1_ V_1_) [example (5) in Table S4], which is in the canonical word order for Kazakh, and ii) the main clause followed by the subordinate clause [example (7) in Table S4]; these two types were presented in a randomized order. The G3 condition, thus, could not be correctly executed with knowledge of word sequences alone. In Kazakh, the personal suffix of a perfect adjectival participle (e.g., V_2_) in a *subordinate* clause is always fixed irrespective of the person and number of the subject (see Table S3, verbs). For this special case, it was thus necessary to abandon the subject-verb agreement rules once acquired through G1 and G2. Through testing with these G1-G3 conditions, participants progressively acquired certain syntactic features of Kazakh, more specifically, the syntactic structures of sentences in Kazakh.

***Language proficiency assessment by Avant tests***

The language proficiency of all the participants was assessed by the Avant STAMP 4S (Standards-based Measurement of Proficiency - 4 Skills; Avant Assessment, Eugene, OR, USA). The Avant STAMP 4S is an online, proficiency-oriented assessment of the four skills of listening, reading, writing, and speaking for 14 languages; we used only the listening skill tests in this study, because we were focused on listening proficiency in Kazakh. According to the American Council on the Teaching of Foreign Languages (ACTFL) proficiency guidelines 2012, results of each *adaptive* test are graded by scores 1-9: Novice (1-3), Intermediate (4-6), and Advanced (7-9), where identical scores for different languages estimated comparable proficiency levels among languages. The Avant test in English was administered to all participants. As regards other languages, participants first received a sample Avant test (Spanish, Russian, German, Chinese, Italian, or Korean), and those who were able to answer the first question in this sample test then received the full test. If a participant stopped taking the sample test, the score was regarded as none.

***Participants***

Most participants acquired English as their L2 relatively late (age of acquisition (AOA): Bi, 11.1 ± 2.8 years; Multi, 9.8 ± 3.0 years). Only one participant of the Bi group had a history of residence in an English-speaking region (Table S2). Regarding the Multi group, most participants acquired Spanish as an L3 (23 participants) or as an L4 (two participants), whereas five participants had acquired Portuguese, French, Russian, or German as an L3; they had a history of residence outside Japan (16 participants in Spanish-speaking regions, three in English-speaking regions, and five for both). Although proficiency in a language is generally influenced by the frequency of exposure and usage of the language (e.g., intense use while living abroad) even for multilinguals or bilinguals, the development of these communicative abilities and/or pragmatic knowledge was irrelevant to the present study. The language acquired as an L2 by the participants, and those acquired as an L3 by the multilinguals, are all *head-initial* languages with a subject-verb agreement. In contrast, Japanese and Kazakh are *head-final* languages, but Japanese lacks a subject-verb agreement. Therefore, the variation in L3 would not affect the present Kazakh experiments on this respect. For the two participants, whose L4 was Spanish, only a Spanish version of the Avant test was administered; Spanish represented their L3 for the analyses of Fig. 2, Fig. 7, and Table S1.

***Stimuli***

Each trial began with a visual cue indicating one of the *conditions* Words (W), G1, G2, and G3 (see Fig. 1), followed by the “Lexical list” of five words used for that trial. The Lexical list consisted of two verbs, two nouns (a pronoun and a proper noun), and one of the four function words, always in this order. Because the verbs we used were generally longer than the nouns, we adjusted the duration of a verb and that of a noun to 0.6 s and 0.5 s, respectively, maintaining the original pitch for each word. For the Lexical list, *auditory* words in Kazakh were accompanied by *visual* words in English (a translation of each Kazakh word; verbs were displayed for 1.1 s, other words for 1.0 s). When auditory stimuli were presented with visual stimuli, the visual stimuli was shown 0.25 s earlier. The English word presentation (with an interval of 0.5 s) served as a minimal reference for recognition of meanings and parts of speech in Kazakh. This translation was presented at the start of every trial, and, hence, the participants did not have to keep these translations in memory for the subsequent trials. Within a trial, the English translation was presented for the Lexical list alone.

During the scans, the participants wore an eyeglass-like MRI-compatible display (resolution = 800×600 pixels, framerate = 60 fps; VisuaStim Digital, Resonance Technology Inc., Northridge, CA). A small red cross was always shown at the center of the screen, and the participants were instructed to fixate on it as much as possible during the tasks. The stimulus presentation and collection of behavioral data (response times (RTs) and accuracy) were controlled using the Presentation software package (Neurobehavioral Systems, Albany, CA).

***Tasks***

In each demo trial under the Words condition (Fig. 1a, left), a Lexical list was followed by a set of five words (“Lexicons”), which was auditorily presented and compared with the five words from the Lexical list. For the Lexical list and Lexicons, the same lexical items were presented in the same order, but with some inflectional changes. During the presentation of the Lexicons, a row of five signs [either plus (+) or minus (−) sign] was displayed as correctness of matching. Each sign from left to right represented the matched (+) or unmatched (−) status of each word of the Lexicons in terms of inflection. If the Lexicons included one of the function words *al*, *dep*, *kezde*, and *adam/adamdï*, the total duration of the Lexicons was adjusted to 4.1 s, 4.2 s, 4.35 s, and 4.35 s, respectively.

With respect to a task trial under the Words condition (Fig. 1a, right), the Lexical list and the Lexicons were presented in the same manner as the demo trials without the +/− signs, followed by a lexical matching task (“W Task”). During the W Task, while visually presented with the symbols 1, 2, 3, and 4 for the four buttons, the participants pressed a button within 3.0 s, according to the number of *unmatched* words between the Lexical list and the Lexicons in the same trial (comparable to the number of *minus* signs in a demo trial).

In each demo trial under the Grammar conditions (Fig. 1b, left), the Lexical list always had nouns presented in the nominative case, and verbs presented in the past tense without a personal suffix (see Table S3). This Lexical list was followed by a five-word sentence (“Sentence”) using all of the five words presented in the Lexical list. During the temporal event of the Sentence, a visual sign was simultaneously presented, representing the correctness (i.e., grammaticality) of the sentence (see Table S4), either grammatical (+) or ungrammatical (−). An ungrammatical sentence always included an error of verb inflection necessary for the subject-verb agreement. After a brief pause of 2.7 s, a “Noun-verb pair” taken from the Sentence was auditorily presented with a pause of 1 s between the noun and verb, together with a visual sign (for 2.7 s). This sign represented the correctness of the Noun-verb pairing, either correct (+; N_1_ V_1_ or N_2_ V_2_) or incorrect (−; N_1_ V_2_ or N_2_ V_1_) matching. In this pair, the nouns were always presented in the nominative case, and the verbs were always presented in the past tense without a personal suffix.

With respect to the task trial under the Grammar conditions (Fig. 1b, right), the Lexical list, Sentence, and Noun-verb pair were presented similarly to the demo trials without signs, where a grammaticality judgement task (“GR Task”) and an subject-verb matching task (“SV Task”) followed the Sentence and Noun-verb pair, respectively. In a GR Task, the participants judged whether the Sentence was grammatical or not, and pressed one of the two buttons within 3.0 s, while symbols of the two *left* buttons (see Fig. 1b) were visually presented (+ and −; comparable to the plus or minus sign in a demo trial). In the SV Task, the participants judged whether the Noun-verb pair correctly matched with an subject-verb in the Sentence or not, and pressed one of the two buttons within 3.0 s, while symbols of the two *right* buttons (see Fig. 1b) were visually presented (+ and −; comparable to the plus or minus sign in a demo trial). The criterion for mastering each of the W, GR, and SV Tasks was to perform correctly in at least six out of the eight task trials in each of two blocks (not necessarily consecutive). In order to finish each of the G1-G3 conditions (see Table S4), this criterion must have been met for both of the GR and SV Tasks in the same two blocks. We measured RTs from the onset of the W, GR, or SV Task.

During the temporal events of the *Sentence* under the Grammar conditions, syntactic structures of a sentence in Kazakh, subject-verb agreement, and grammaticality were evaluated (see Tables S3 and S4), and were tested immediately afterwards using the GR and SV Tasks. The *Lexical lists* were physically identical between the Words and Grammar conditions, but had different roles. Under the Words condition, the Lexical list of the Kazakh words had to be memorized, while under the Grammar conditions, this list was shown just for confirmation of the words of a sentence in the same trial. During the temporal events of the *Lexicons* under the Words condition, the precise identification of sounds of the Kazakh words, recall of words in the Lexical list, and matching those words were involved for the W Task. Therefore, the Lexical lists controlled common basic auditory and encoding processes against the Sentence, whereas the Lexicons strictly controlled auditory and memory retrieval processes as well.

The participants first engaged in trials under the Words condition (Wpre), which were *preparatory* to the Grammar conditions. For these preparatory trials outside the scanner, the Lexical list contained nouns in the nominative case, and verbs in the past tense without a personal suffix. By using these preparatory processes, the inflectional changes for the Lexicons, which were necessary to understand the subject-verb agreement under the Grammar conditions, were acquired. Once the participants mastered the W Task through the Wpre blocks, they proceeded to the scanning sessions under the Grammar conditions, in the order of G1, G2, and G3.

Under the Grammar conditions, demo trials were always conducted without MR scanning in a silent environment. The four demo trials in a block consisted of three grammatical sentences and one ungrammatical sentence for the Sentence, as well as three correct pairs and one incorrect pair for the Noun-verb pair. The ungrammatical sentence and the incorrect pair did not appear in the same trial, and each of them appeared separately in the last two trials of the block. In an MR scanning *run* with task trials, three Words trials, a block of eight Grammar trials, and two Words trials (i.e., 13 *task trials* in total) were tested in this order; i.e., the target Grammar trials interwoven between the control Words trials. The function words appearing in the control Words trials matched those in the target trials. For the control Words trials during G1 and G2 runs, the Lexical list had nouns in the nominative case, and verbs in the past tense without a personal suffix, just as in Wpre. For those Words trials during the more advanced G3 runs, the nouns and verbs in the Lexical list could have inflectional changes in our experiment (see above). Before starting the G3 runs, the participants received demo and task trials for the new Words trials, until they met the criterion for a single block outside the scanner.

Before starting each Grammar condition, outside the MRI scanner, we provided the participants with an English translation of a typical Kazakh sentence used in that condition. Before the day's initial session, the participants reviewed exercises outside the scanner, until they met the criterion (only in one block for this reviewing) under every condition they had reached previously. Multiple sessions in a day were conducted for less than 2.5 hours, including instructions and a few breaks; a break was inserted at least after seven consecutive runs under each Grammar condition. All sessions were conducted in a span of two to four days; inter-session intervals were less than four weeks, with one exception (37 days).

***fMRI data analyses***

The acquisition timing of each slice was corrected using the middle slice (the 15th slice chronologically) as a reference for the functional images. We spatially realigned each volume to the first volume of consecutive runs, and a mean volume was obtained. We set the threshold of head movement during a single run: within a displacement of 2 mm in any of the three directions, and within a rotation of 1.4˚ around any of the three axes. These thresholds were empirically determined from our previous studies ^1^. If a run included one or several images over this threshold, we replaced the outlying image with an interpolated image, which was the average of the chronologically former and latter ones, and conducted the realignment procedure again; data under the G3 condition for one participant from the Multi group was excluded from both activation and behavioral analysis, due to excessive head movement. The realigned data were resliced every 3 mm using seventh-degree B-spline interpolation.

Each individual’s structural image was matched with the mean functional image generated during realignment. The resultant structural image was spatially normalized to the standard brain space as defined by the Montreal Neurological Institute (MNI) using the extended version of unified segmentation algorithm with light regularization; this is a generative model that combines tissue segmentation, bias correction, and spatial normalization in a single model ^2^. The resultant deformation field was applied to each realigned functional image to be spatially normalized with non-linear transformation. All normalized functional images were then smoothed by using an isotropic Gaussian kernel of 9 mm full-width at half maximum (FWHM). Low-frequency noise was removed by high-pass filtering at 1/128 Hz.

In a first-level analysis (i.e., the fixed-effects analysis within a participant), each participant’s hemodynamic responses were modeled for the following types (event types): Lexical list, Lexicons, W Task, Sentence, GR Task, Noun-verb pair, and SV Task (see Fig. 1). The Lexical lists in Grammar and Words trials were regarded as the same type, and these event types were separately set for group [Multi, Bi] and run [G1 first, G1 last, G2 first, G2 last, G3 first, G3 last]. Each event was modeled with the boxcar function overlaid with a hemodynamic response function. To minimize the effects of head movement, the six realignment parameters obtained from preprocessing were included as a nuisance factor in a general linear model.

These modeled responses were then generated in a general linear model for each participant and used for the inter-subject comparison in a second-level analysis (i.e., the random-effects analysis for a group). To examine the activation of the regions in an unbiased manner, we adopted whole-brain analyses. For functional analyses with *t*-tests, a one-way repeated measures analysis of covariance (rANCOVA) on event type (Fig. 5), a two-way rANCOVA [run × event type] within a group (Fig. 4ab), and a three-way rANCOVA [group × run × event type] (Fig. 4c), were performed with three nuisance factors (age, gender, and laterality quotient). For the third analysis which spanned multiple Grammar conditions, the participants who did not meet passing criteria for these multiple conditions were dropped. For the anatomical identification of activated regions, essentially we used the Anatomical Automatic Labeling (AAL) method (http://www.gin.cnrs.fr/AAL2/) ^3^ and the labeled data as provided by Neuromorphometrics Inc. (http://Neuromorphometrics.com/) under academic subscription. In addition to whole-brain analyses, we adopted analyses of each region of interest (ROI) by using AAL masks and the MarsBaR-toolbox (http://marsbar.sourceforge.net/). The ROI of the Basal Ganglia/Thalamus consisted of four regions (Caudate, Putamen, Pallidum, and Thalamus), that of the Cerebellum VI/Crus I consisted of two regions (Cerebelum_VI and Cerebelum_Crus I), that of the MFG/IFG consisted of five regions (Frontal_Inf_Oper, Frontal_Inf_Tri, Frontal_Inf_Orb, Frontal_Mid, and Precentral), that of the STG/MTG consisted of five regions (Temporal_Sup, Temporal_Pole_Sup, Temporal_Mid, Temporal_Pole_Mid, and Heschl), and that of the Calcarine/LG consisted of two regions (Calcarine and Lingual). Because signal changes and number of voxels within a ROI yield comparable results or those with one-sided dominance depending on the variance among individuals ^4,5^, we adopted the voxel count method for the data with possible group differences.

**References**

1 Kinno, R., Kawamura, M., Shioda, S. & Sakai, K. L. Neural correlates of noncanonical syntactic processing revealed by a picture-sentence matching task. *Hum. Brain Mapp.* **29**, 1015-1027, doi:10.1002/hbm.20441 (2008).

2 Ashburner, J. & Friston, K. J. Unified segmentation. *Neuroimage* **26**, 839-851, doi:10.1016/j.neuroimage.2005.02.018 (2005).

3 Tzourio-Mazoyer, N. *et al.* Automated anatomical labeling of activations in SPM using a macroscopic anatomical parcellation of the MNI MRI single-subject brain. *Neuroimage* **15**, 273-289, doi:10.1006/nimg.2001.0978 (2002).

4 Chee, M. W. L., Lee, H. L., Soon, C. S., Westphal, C. & Venkatraman, V. Reproducibility of the word frequency effect: Comparison of signal change and voxel counting. *Neuroimage* **18**, 468-482, doi:10.1016/s1053-8119(02)00019-8 (2003).

5 Bhattacharyya, P. K., Phillips, M. D., Stone, L. A. & Lowe, M. J. Activation volume vs BOLD signal change as measures of fMRI activation – Its impact on GABA – fMRI activation correlation. *Magn. Reson. Imaging* **42**, 123-129, doi:10.1016/j.mri.2017.06.009 (2017).
